# Hepatitis C Virus Core Mutations Associated with False-Negative Serological Results for Genotype 3a Core Antigen

**DOI:** 10.1128/JCM.01062-15

**Published:** 2015-07-20

**Authors:** Linh Thuy Nguyen, Linda Dunford, Ines Freitas, Paul Holder, Lan Anh Nguyen, Joanne O'Gorman, Jeff Connell, Michael Carr, William Hall, Cillian De Gascun

**Affiliations:** a Ireland Vietnam Blood-Borne Virus Initiative, Dublin, Ireland, and Hanoi, Vietnam; b National Virus Reference Laboratory, University College Dublin, Belfield, Ireland; c Centre for Research in Infectious Diseases, School of Medicine and Medical Science, University College Dublin, Belfield, Ireland; d Laboratory for Molecular Diagnostics, National Institute of Hygiene and Epidemiology, Hanoi, Vietnam

## Abstract

Genetic characterization of the genotype 3a (GT3a) hepatitis C virus (HCV) *core* region from HCV core antigen (HCVcAg)-negative/RNA-positive cases and HCVcAg-positive/RNA-positive controls identified significant associations between the substitutions A48T and T49A/P and failure to detect HCVcAg (*P* < 0.05). Polymorphisms at residues 48 and 49 in the core protein are present across all major epidemic and endemic GTs. These findings have implications for HCV diagnosis, particularly in low-income regions in which GT3a HCV is endemic.

## TEXT

Hepatitis C virus (HCV) is a global pathogen, infecting more than 185 million individuals, with global seroprevalence estimated at 2.8% (95% uncertainty interval [UI], 2.6 to 3.1%) and more than 1 million new cases reported annually (1). Previous studies on the detection of HCV core antigen (HCVcAg) demonstrated that this antigen (Ag) represents a robust stable marker of HCV replication (2). Quantification of HCVcAg can be performed using an automated, highly reproducible, chemiluminescent, microparticle immunoassay with a shorter turnaround time and lower costs than quantitative real-time PCR (qPCR) (3). However, the assay is less sensitive than qPCR in detecting viremia and has not been recommended for monitoring responses to antiviral therapy (4). The Architect HCV Ag assay has shown good correlation of HCVcAg and HCV RNA measurements irrespective of the HCV genotype (GT) (2, 5), although there are reports that the correlation of HCVcAg and RNA measurements is more robust for GT1 and GT4 than for GT3 (6), with greater variance for positive GT3 samples than for samples of other GTs (7). However, the cause of those discordances was not explored in those studies. The present study was undertaken to investigate the discordance between HCVcAg and RNA measurements in GT3a HCV-infected individuals.

Plasma/serum samples from HCV-infected individuals (*n* = 511) that were referred to the National Virus Reference Laboratory (NVRL) for HCV investigations were included to correlate HCVcAg and RNA measurements for GT1a, GT1b, and GT3. False-negative HCVcAg cases were defined as cases with undetectable HCVcAg but HCV RNA levels of ≥4 log_10_ IU/ml, which is above the lower limit of detection of the HCVcAg assay, based on previous studies (7–9) and our own analyses. The controls were samples in which both HCVcAg and RNA were detectable. HCVcAg was quantified on the Architect HCV Ag platform (Abbott Diagnostics, Wiesbaden, Germany). The assay cutoff threshold for a positive result is ≥3 fmol/liter, whereas values of 3 to 10 fmol/liter and >10 fmol/liter are reported as weak positive and positive, respectively. HCV viral loads were determined using the Abbott Molecular m2000 RealTime System (Abbott Diagnostics, Wiesbaden, Germany). HCV genotyping was performed with the Innogenetics Versant HCV GT2.0 assay (Siemens Healthcare, Milan, Italy) or the RealTime HCV Genotype II assay (Abbott, Wiesbaden, Germany). Employing a previously described method (10), a 1,256-bp fragment encompassing the entire HCV *core* gene from the 5′ untranslated region (UTR) to the *E1* gene was amplified for bidirectional sequencing, which was performed on the 3500 Dx platform (Applied Biosystems, Foster City, CA) using BigDye Terminator chemistry (version 3.1). Sequence chromatograms were investigated manually using sequence analysis software (SeqMan Pro version 11.2.1; DNAStar). The consensus sequences were aligned by using ClustalW in Bioedit (version 7.05). Fisher's exact test (categorical variables), the Mann-Whitney *U* test (continuous variables), and Spearman's rank correlation coefficient tests were performed using MedCalc version 14.8.1.

**FIG 1:**
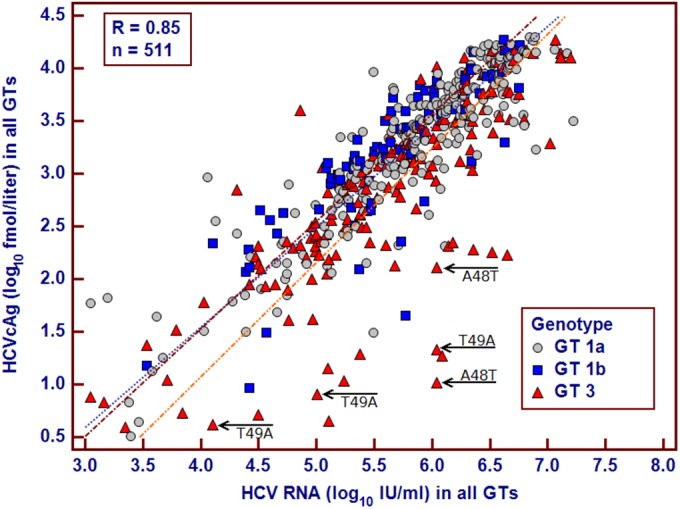
Scatter plots depicting the correlation between HCVcAg (log_10_ fmol/liter) and HCV RNA (log_10_ IU/ml) measurements. Arrows, samples in which A48T (*n* = 2) and T49A (*n* = 3) were identified in the GT3a HCV core protein.

HCVcAg- and RNA-positive samples for GT1a (*n* = 261), GT1b (*n* = 79), and GT3 (*n* = 171) were evaluated. Strong positive correlations between HCVcAg and RNA levels (*P* < 0.0001) were observed for all three GTs (Fig. 1). However, the correlation coefficient for the correlation of HCVcAg and RNA levels was lower for GT3 (*r* = 0.79) than for GT1a (*r* = 0.87) and GT1b (*r* = 0.86). Greater variance for GT3, compared to GT1a and GT1b, was apparent (Fig. 1). Furthermore, the HCVcAg/RNA ratio for GT3 was significantly lower than those for GT1a and GT1b (*P* < 0.0001), while there was no significant difference in the HCVcAg/RNA ratios for GT1a and GT1b (*P* = 0.44), indicating the underquantification of HCVcAg, relative to the corresponding viral loads, for GT3 samples.

**FIG 2:**
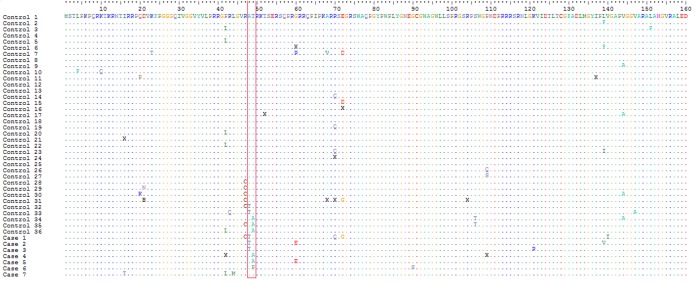
Amino acid sequence alignment (160 residues) of the mature GT3a HCV core protein, showing comparison of sequences derived from controls (*n* = 36) and cases (*n* = 7). Amino acid positions in the HCV core protein are numbered, and sequence identity is represented by single dots. Red box, region including residues 48 and 49, which were significantly associated with compromised HCVcAg measurements. X, presence of more than one amino acid.

Genetic characterization of the *core* gene in GT3a HCV false-negative cases and controls determined amino acid substitutions associated with the underquantification of HCVcAg. A48T was found in 5.5% of controls (2/36 samples) versus 42.9% of cases (3/7 samples) (*P* < 0.05). T49A/P was found in 8.3% of controls (3/36 samples) versus 42.9% of cases (3/7 samples) (*P* < 0.05). The alignment of 160 amino acid residues in the core region of 36 controls and seven cases is shown in Fig. 2. For case 7, substitutions at residues 48 and 49 were absent, but we noted the presence of L44M; this mutation was not seen in other sequences from either cases or controls (Fig. 2). In the Los Alamos database, L44 predominates in all HCV GTs (range, 98.58 to 100%) and is present in 100% of GT3a sequences, which indicates the relative rarity of L44M. Consequently, this mutation may also affect the ability of the monoclonal antibodies in the assay to detect HCVcAg.

Additionally, five controls with substitutions in either residue 48 (*n* = 2) or residue 49 (*n* = 3) (Fig. 1) deviated significantly from the trend of the majority of GT3 samples, and their HCVcAg/RNA ratios were significantly lower than those for the other controls, without these substitutions (*P* = 0.0009). The characteristics of controls and cases containing significant substitutions are shown in Table 1. These data suggest that underquantification of HCVcAg levels can occur when substitutions at residues 48 and 49 are present in the HCV core protein.

**TABLE 1:** Statistically significant substitutions at positions 48 and 49 in GT3a HCV core antigen associated with discordant HCV RNA and HCVcAg measurements for HCVcAg-suppressed controls (*n* = 5) and HCVcAg-negative cases (*n* = 7)

Sample	Amino acids^*a*^		HCV RNA level (log_10_ IU/ml)	HCVcAg level (fmol/liter)	HCVcAg status
	Position 48	Position 49			
Control 32	**A48T**	T49	6.04	10.5	Positive
Control 33	**A48T**	T49	6.04	128.8	Positive
Control 34	A48	**T49A**	6.04	21.4	Positive
Control 35	A48	**T49A**	5.01	8.1	Weak positive
Control 36	A48	**T49A**	4.10	4.2	Weak positive
Case 1	**A48T**	T49	5.00	<3	Negative
Case 2	**A48T**	T49	5.37	<3	Negative
Case 3	**A48T**	T49	5.38	<3	Negative
Case 4	A48	**T49A**	5.03	<3	Negative
Case 5	A48	**T49A**	4.35	<3	Negative
Case 6	A48	**T49P**	5.07	<3	Negative
Case 7	A48	T49	5.42	<3	Negative

a Amino acid substitutions are shown in bold.

To evaluate the degree of conservation of the HCV core protein, all available core sequences (*n* = 5,623) were downloaded from the Los Alamos database (http://hcv.lanl.gov/content/index) and aligned with the Web-based Jalview (11). At position 48, alanine is the most common residue, while A48T is present in 0.35 to 6.70% of all GTs. At position 49, threonine predominates; however, T49A was seen in all GTs (range, 0.1 to 4.5%). T49P is also present in GT1a, GT1b, GT2, and GT4 (range, 0.4 to 15.7%). Overall, in GT3a, A48T was seen in 2 (0.71%) of 282 deposited sequences; these two sequences originated in Sweden and India. T49A was seen in 12 (4.26%) of 282 GT3a sequences; six of these sequences originated in Pakistan, three were from China, Thailand, and the United States, and the other three had no information on the country of origin.

In 2000, Tokita et al. identified 4% of GT1b HCV samples (4/107 samples) with relatively low HCVcAg levels, as measured with a fluorescence enzyme immunoassay, compared to corresponding HCV RNA values (12). All four samples showed a point mutation (T49P) in comparison with eight controls in which this substitution was not found. In 2012, a German group reported one GT3 HCV case with an HCVcAg level of only 5.16 fmol/liter, as detected with the Abbott Architect assay, despite a viral load of 5.63 log_10_ IU/ml (B. Schulte, S. Susser, B. Ritter, C. Sarrazin, A. Heim, and B. Wolk, presented at an Abbott-sponsored symposium, 2012). Two mutations in an epitope targeted by monoclonal antibodies in the HCVcAg assay were identified; however, neither the epitope nor the substitution was specified in the presentation. The authors stated that the core sequence analysis from the European Hepatitis C Virus Database showed that the coincidence of these mutations was infrequent. In another study, Murayama et al. evaluated the correlation of HCV RNA and HCVcAg measurements with five different commercial HCVcAg assays, using a reference panel of GT1 and GT2 samples (13). Twelve GT1b or GT2 samples exhibited the polymorphisms R47G, A48T, and T49A/P, which correlated with HCVcAg underquantification with multiple HCVcAg assays. Those results showed that the Architect assay exhibited the highest sensitivity; however, generally the sensitivity of all of the commercially available HCVcAg assays was still insufficient to detect low-titer HCV infections (13). The false-negative HCVcAg results in the present study were associated with low to medium viral loads (range, 4.35 to 5.42 log_10_ IU/ml), while our data suggested that 42.8% of HCV-positive samples (219/511 samples) had high viral loads (≥6 log_10_ IU/ml). The Architect HCVcAg assay has proved invaluable in the diagnosis and management of HCV infections. The assay facilitates the detection of viremic individuals without the need for HCV RNA investigations, allowing more rapid referral and risk management. The test is also useful for the identification of individuals with spontaneously resolved HCV infections. While a false-negative HCVcAg result could indicate erroneously that an infection had resolved, current best practices would ensure HCV RNA testing to confirm this interpretation. Finally, the HCVcAg test has provided a logistically easier method to screen for the presence of acute HCV infections in the settings of dialysis units, organ donor assessments, percutaneous injuries, or relapses following HCV treatment. In these scenarios, it is probable that HCV viral loads would be sufficiently high for the HCVcAg assay to be employed for HCV detection.

Notably, after GT1, GT3 is second most prevalent GT worldwide (54.3 million cases [30.1% of the total]), and its seroprevalence is highest in southern Asia (14). This distribution has been attributed potentially to the association of GT3 with persons who inject drugs (15) and recent migrations from India and Pakistan, where GT3a predominates (15–17). Therefore, the endemic nature of GT3 HCV in southern Asia might have implications for the utilization of the HCVcAg assay for detection of acute infections. The inclusion of confirmatory protocols in the management of HCV infections is indicated, particularly in low-income regions in which access to qPCR assays is limited and GT3a HCV is endemic.

### Nucleotide sequence accession numbers.

The sequences determined were submitted to GenBank with the following accession numbers: KP797837 to KP797872 (controls) and KP797873 to KP797879 (cases).

## References

[1] Mohd Hanafiah K, Groeger J, Flaxman AD, Wiersma ST 2013 Global epidemiology of hepatitis C virus infection: new estimates of age-specific antibody to HCV seroprevalence. Hepatology 57:1333–1342. doi:10.1002/hep.26141. 23172780

[2] Mederacke I, Wedemeyer H, Ciesek S, Steinmann E, Raupach R, Wursthorn K, Manns MP, Tillmann HL 2009 Performance and clinical utility of a novel fully automated quantitative HCV-core antigen assay. J Clin Virol 46:210–215. doi:10.1016/j.jcv.2009.08.014. 19766055

[3] Morota K, Fujinami R, Kinukawa H, Machida T, Ohno K, Saegusa H, Takeda K 2009 A new sensitive and automated chemiluminescent microparticle immunoassay for quantitative determination of hepatitis C virus core antigen. J Virol Methods 157:8–14. doi:10.1016/j.jviromet.2008.12.009. 19135481

[4] Heidrich B, Pischke S, Helfritz FA, Mederacke I, Kirschner J, Schneider J, Raupach R, Jackel E, Barg-Hock H, Lehner F, Klempnauer J, von Hahn T, Cornberg M, Manns MP, Ciesek S, Wedemeyer H 2014 Hepatitis C virus core antigen testing in liver and kidney transplant recipients. J Viral Hepat 21:769–779. doi:10.1111/jvh.12204. 24251818

[5] Chevaliez S, Soulier A, Poiteau L, Bouvier-Alias M, Pawlotsky JM 2014 Clinical utility of hepatitis C virus core antigen quantification in patients with chronic hepatitis C. J Clin Virol 61:145–148. doi:10.1016/j.jcv.2014.05.014. 24973282

[6] Garbuglia AR, Monachetti A, Galli C, Sabatini R, Ferreri ML, Capobianchi MR, Bagnarelli P 2014 HCV core antigen and HCV-RNA in HIV/HCV co-infected patients with different HCV genotypes. BMC Infect Dis 14:222. doi:10.1186/1471-2334-14-222. 24758157 PMC4029812

[7] Ottiger C, Gygli N, Huber AR 2013 Detection limit of Architect hepatitis C core antigen assay in correlation with HCV RNA, and renewed confirmation algorithm for reactive anti-HCV samples. J Clin Virol 58:535–540. doi:10.1016/j.jcv.2013.08.028. 24041472

[8] Ross RS, Viazov S, Salloum S, Hilgard P, Gerken G, Roggendorf M 2010 Analytical performance characteristics and clinical utility of a novel assay for total hepatitis C virus core antigen quantification. J Clin Microbiol 48:1161–1168. doi:10.1128/JCM.01640-09. 20107102 PMC2849592

[9] Medici MC, Furlini G, Rodella A, Fuertes A, Monachetti A, Calderaro A, Galli S, Terlenghi L, Olivares M, Bagnarelli P, Costantini A, De Conto F, Sainz M, Galli C, Manca N, Landini MP, Dettori G, Chezzi C 2011 Hepatitis C virus core antigen: analytical performances, correlation with viremia and potential applications of a quantitative, automated immunoassay. J Clin Virol 51:264–269. doi:10.1016/j.jcv.2011.05.003. 21621454

[10] Dunford L, Carr MJ, Dean J, Waters A, Nguyen LT, Ta Thi TH, Thi LA, Do HD, Thi TT, Nguyen HT, Diem Do TT, Luu QP, Connell J, Coughlan S, Hall WW, Nguyen Thi LA 2012 Hepatitis C virus in Vietnam: high prevalence of infection in dialysis and multi-transfused patients involving diverse and novel virus variants. PLoS One 7:e41266. doi:10.1371/journal.pone.0041266. 22916104 PMC3419252

[11] Waterhouse AM, Procter JB, Martin DM, Clamp M, Barton GJ 2009 Jalview version 2: a multiple sequence alignment editor and analysis workbench. Bioinformatics 25:1189–1191. doi:10.1093/bioinformatics/btp033. 19151095 PMC2672624

[12] Tokita H, Kaufmann RG, Matsubayashi M, Okuda I, Tanaka T, Harada H, Mukaide M, Sujuki K, Cooper AD 2000 Hepatitis C virus core mutations reduce the sensitivity of a fluorescence enzyme immunoassay. J Clin Microbiol 38:3450–3452. 10970401 10.1128/jcm.38.9.3450-3452.2000 PMC87404

[13] Murayama A, Sugiyama N, Watashi K, Masaki T, Suzuki R, Aizaki H, Mizuochi T, Wakita T, Kato T 2012 Japanese reference panel of blood specimens for evaluation of hepatitis C virus RNA and core antigen quantitative assays. J Clin Microbiol 50:1943–1949. doi:10.1128/JCM.00487-12. 22495557 PMC3372108

[14] Messina J, Humphreys I, Flaxman A, Brown A, Cooke G, Pybus OG, Barnes E 2015 Global distribution and prevalence of hepatitis C virus genotypes. Hepatology 61:77–87. doi:10.1002/hep.27259. 25069599 PMC4303918

[15] Pybus OG, Cochrane A, Holmes EC, Simmonds P 2005 The hepatitis C virus epidemic among injecting drug users. Infect Genet Evol 5:131–139. doi:10.1016/j.meegid.2004.08.001. 15639745

[16] Roman F, Hawotte K, Struck D, Ternes A, Servais J, Arendt V, Hoffman P, Hemmer R, Staub T, Seguin-Devaux C, Schmit J 2008 Hepatitis C virus genotypes distribution and transmission risk factors in Luxembourg from 1991 to 2006. World J Gastroenterol 14:1237–1243. doi:10.3748/wjg.14.1237. 18300350 PMC2690672

[17] Uddin G, Shoeb D, Solaiman S, Marley R, Gore C, Ramsay M, Harris R, Ushiro-Lumb I, Moreea S, Alam S, Thomas HC, Khan S, Watt B, Pugh RN, Ramaiah S, Jervis R, Hughes A, Singhal S, Cameron S, Carman WF, Foster GR 2010 Prevalence of chronic viral hepatitis in people of south Asian ethnicity living in England: the prevalence cannot necessarily be predicted from the prevalence in the country of origin. J Viral Hepat 17:327–335. doi:10.1111/j.1365-2893.2009.01240.x. 20002307

